# Differential Expression of miRNA Regulates T Cell Differentiation and Plasticity During Visceral Leishmaniasis Infection

**DOI:** 10.3389/fmicb.2016.00206

**Published:** 2016-02-25

**Authors:** Rajan Kumar Pandey, Shyam Sundar, Vijay Kumar Prajapati

**Affiliations:** ^1^Department of Biochemistry, School of Life Sciences, Central University of RajasthanKishangarh, India; ^2^Department of Medicine, Institute of Medical Sciences, Banaras Hindu UniversityVaranasi, India

**Keywords:** MicroRNAs, *Leishmania donovani*, visceral leishmaniasis, Th1/Th2, CD4^+^ T cell, Th17/Treg

## Abstract

Visceral leishmaniasis (VL) is a tropical neglected disease caused by *Leishmania donovani*, results in significant mortality in the Indian subcontinent. The plasticity of T cell proliferation and differentiation depends on microRNA mediated gene regulation which leads Th1/Th2 or Th17/Treg type of immune response during human VL. This study depicts the identification of target immune signaling molecule and transcription factors, which play a role in T-cell proliferation and differentiation followed by the identification of miRNA controlling their gene expression using three web servers’ viz., TargetScan, miRPath and miRDB. This study provides the bioinformatics evidences that seed region present in the miRNAs miR-29-b, miR-29a, have the putative binding site in the 3′-untranslated region (UTR) of TBX21 transcription factor of CD4^+^ T helper (Th1), which may suppress the Th1 specific protective immune response. Development of Th2 type specific immune response can be suppressed by binding of miR-135 and miR-126 miRNAs over the 3′-UTR region of GATA-3 transcription factor of Th2 specific CD4^+^ T helper cells. MiRNA identified against Th2/Treg immune cells are important and their over expression or administration can be used for developing the Th1/Th17 type of protective immune response during VL infection. This study indicates that miRNAs have the capacity to regulate immune signaling, cytokine production and immune cell migration to control the VL infection in human. This observation warrants further investigation for the development of miRNA based therapy controlling T cell differentiation in human VL.

## Introduction

Leishmaniasis is a tropical neglected diseases which affect 350 million people worldwide living in 98 countries and three territories of five continents (Alvar et al., 2012). The increasing incidence of leishmaniasis show different faces such as visceral, cutaneous and muco-cutaneous and post kala-azar dermal leishmaniasis. Among all these form of leishmaniasis, visceral leishmaniasis (VL) is most severe which affects 58,200 individuals yearly worldwide. It causes absolute death if untreated and is clearly a poverty-related disease which accounts over 30,000 deaths annually (Prajapati et al., 2012). During VL infection, serum plays an important role in binding of promastigotes to macrophage through adsorption of opsonins (antibody/complement protein) to the parasite and subsequent binding to Fc receptor of the macrophage followed by endocytosis (Sharma and Singh, 2009). Therefore, parasites escape the humoral immune response of host by residing in the phagolysosome of macrophage and subsequently antibodies have no effect on the infection which may be harmful to the host (Unanue and Allen, 1987).

*Leishmania donovani* infection and its presentation by conventional antigen presenting cells induce different pro-inflammatory cytokine response. Naive CD4^+^ T cell can initiates differentiation into specific lineages such as Th1, Th2, Th17 and regulatory T (Treg) cells depending upon pro-inflammatory response and expression of specific transcription factors. Each T cell lineage has distinct effectors mechanism along with specific cytokine profiles. In response of IFN-γ and IL-12 pro-inflammatory cytokines, naïve CD4^+^ T cells differentiate into Th1 cell through controlled process and activity of STAT1, STAT4 and T-bet transcription factors. The STAT4 transcription factor is required to initiate IL-12 signaling along with IFN-γ and inducible nitric oxide synthase expression. Th1 immune cells are the main player of parasite clearance during VL by producing a large amount of IFN-γ and play a critical role to protect human against intracellular parasite by the activation of macrophages (Romagnani, 1999). On the other way, IL-4 promotes the differentiation of naïve CD4^+^ T cell into Th2 type immune cell through the activation of STAT6 and transcription factor GATA3. In case of VL, Th2 cells help in parasite survival by down regulation of IL-12 via IL-4, to maintain the homeostasis between Th1 and Th2 (Sundar et al., 1997). The differentiation of CD4^+^ T cells to Th17 cells is carried out in response to IL6 and TGF-β. Initially, IL-6 activates STAT3, then the combined effect of IL-6 and TGF-β signaling leads to the expression of retinoid related orphan receptor (ROR-γt and ROR-α) transcription factor resulting in the initiation of Th17 differentiation. During VL, *L. donovani* induces differentiation of Th17 cells and the resulting IL-17 and IL-22 cytokines are associated with resistance to VL (Pitta et al., 2009). Treg cells differentiation requires same TGF-β to promote the expression of transcription factor Foxp3 but in the absence of IL-6, blocks the expression of ROR-γt and ROR-α transcription factor which results in differentiation of naïve T cell into Treg cells (Zhou et al., 2008).

MiRNAs are evolutionary conserved, non-protein coding, small silencing RNA with size ranges from 20 to 24 nucleotides. The seed sequence of miRNA (7–8 nucleotide) makes base pair with 3′-untranslated region (UTR) of target mRNA results in either translation inhibition or/and mRNA degradation (Bartel, 2009). Till date, several miRNAs have shown the capacity to regulate biological processes like cell differentiation, proliferation, and apoptosis (Miska, 2005; Ruan et al., 2009). It also contributes to the process of thymic T cell maturation and differentiation. The controlled expression of miR-181a contributes to clonal deletion of auto-reactive T cells by the modulation of TCR signaling threshold and survival of low affinity peptide-specific T cell (Li et al., 2007). MiR-155, miR-181c, miR-9, and miR-31 also play an important role in T cell activation by regulating IL-2 signaling pathway. MiR132/212 cluster induces Th17 cells differentiation and its deficiency lowers the frequencies of Th1 and Th17cells due to inhibition of experimental autoimmune encephalitis development (Nakahama et al., 2013). While the over-expression of miR-26a leads to increased Treg expression which play an important role in Th1 and Th17 differentiation (Zhang et al., 2015). In this article, we have presented whether miRNA expression can regulate the CD4^+^ T cell differentiation during VL infection. Here, we have identified transcription factors and important cytokines playing a role in T cell differentiation and favoring the condition for the development of VL disease in human. Using computational study, we predicted up-regulation of putative miRNAs regulating key transcription factor and guiding conversion of Th_0_ cell into Th2 type of immune cells which results in VL infection.

## Materials and Methods

### Identification of Target Immune Signaling Molecules

Visceral leishmaniasis infection in human is associated with activation of Th2 and suppression of Th1 immune response. Suppression of Th1 immune response is associated with decreased level of IFN-γ, IL-12 and higher production of IL-4 and IL-13 cytokines to maintain the Th1/Th2 plasticity. Involvement of Th17 immune cells for the production of IL-17, IL-22 cytokines and to recruit neutrophil is also important factor to provide the protection from VL infection. Development of protective immune response against *L. donovani* parasites depends upon the pro inflammatory immune response which is mediated by antigen presenting cells. In response to pro inflammatory cytokines CD4^+^ T cells converts into either Th1 or Th2 type of the immune cell. Conversion of immune cells in Th1/Th2 is dependent upon the formation of key transcription factors and important cytokines. We have identified key transcription factors and cytokines playing an important role in T cell differentiation and plasticity during the development of protection from VL disease (**Table 1**).

**Table 1:** Key transcription factors regulating CD4+ T cell differentiation and maturation.

S. No.	Name	Category	Function	Reference
1	TBX21	Transcription factor	Induce IFN-γ production	Szabo et al., 2000
	GATA-3	Transcription factor	Master regulator of Th2 cell differentiation	Zheng and Flavell, 1997
2	STAT-1	STAT protein	Induces TBX21 expression	Afkarian et al., 2002
3	STAT-4	STAT protein	Induces IFN-γ production and expression of TBX21	Usui et al., 2003
4	STAT-5	STAT protein	Th2 differentiation	Zhu et al., 2003
5.	STAT-6	STAT protein	Induce GATA-3	Takeda et al., 1996
6	Runx-3/Eomes	Transcription repressor	Induces IFN-γ expression	Naoe et al., 2007
7	IRF-1	IFN regulated factor	Induces IFN-γ expression	Kano et al., 2008
8	IRF-4	IFN regulated factor	Up regulate GATA-3 expression	Lohoff et al., 2002
9	HLX	Other factor	Enhances TBX21 mediated IFN-γ production	Mullen et al., 2002
10	Ets-1	Other factor	Cofactor for TBX21	Grenningloh et al., 2005
11	GFi-1	Transcription repressor	Promote Th2 cell differentiation	Li et al., 1999
12	IKaros	Other transcription factor	Suppress Th1 cell differentiation	Quirion et al., 2009
13	cMaf	Other transcription factor	Enhances IL-4 production	Kim et al., 1999
14	JunB	Other transcription factor	Enhances IL-4 production	Li et al., 1999
15	Dec2	Other transcription factor	Induce GATA-3 expression	Yang et al., 2009
16	Blimp-1	Other transcription factor	Suppresses expression of IL-2 and IFN-γ	Martins and Calame, 2008
17	IL-12	Cytokines	Induction of Th1 cell differentiation	Hsieh et al., 1993
18	IFN-γ	Cytokines	Induction of Th1 cell differentiation	Lighvani et al., 2001
19	IL-4	Cytokines	Induction of Th2 cell differentiation	Swain et al., 1990; Seder et al., 1992
20	IL-2	Cytokines	Induction of Th1 cell differentiation	Le Gros et al., 1990
21	ROR-γt	Transcription factor	Induction of Th17 cell differentiation	Park et al., 2005
22	ROR-α	Transcription factor	Promote Th17 differentiation	Park et al., 2005
23	STAT3	STAT protein	Induction of Th17 cell differentiation	Park et al., 2005
24	IL-6	Cytokine	Induction of Th17 cell differentiation	Harrington et al., 2005
25	TGF-β	Transcription factor	Induction of both Th17and Treg cell differentiation	Bettelli et al., 2006
26	Foxp3	Transcription factor	Induction of Treg cell differentiation	Zhou et al., 2008

### Resources Used for the miRNA Prediction

MiRNAs are small RNA molecule which down regulate the target gene expression at post transcription level. There are many resources available which can be used to predict the most potential miRNAs against target genes. Here, we have tabulated the name of web servers which is freely available for public use and can be used to predict miRNAs (**Table 2**). For this study, we used three web servers viz., TargetScan, miRPath and miRDB to predict miRNAs against key transcription factors and important cytokines regulating CD4^+^ T cell differentiation during VL infection.

**Table 2:** List of resources used for microRNA prediction.

S. No.	Availability	Name of tool	Resource (URL)	Reference
1	Online search	TargetScan	http://www.targetscan.org/	Thomson et al., 2011
2		miRPath	http://diana.imis.athena-innovation.gr/DianaTools/index.php?r~$=$~mirpath/index	Huang et al., 2014
3		miRDB	http://mirdb.org/miRDB/	Wang and El Naqa, 2008
4		miRTrail	http://mirtrail.bioinf.uni-sb.de/mirtrail.php	Yang et al., 2011
5		miRanda	http://www.microrna.org/microrna/home.do	Thomson et al., 2011
6		miRwalk	http://www.umm.uni-heidelberg.de/apps/zmf/mirwalk/	Dweep et al., 2013
7		miRGen	http://carolina.imis.athena-innovation.gr/index.php?r=mirgenv3	Al-Nakhle et al., 2010
8		Pictar	http://pictar.mdc-berlin.de	Thomson et al., 2011

### MiRNAs Prediction Against Transcription Factors Controlling T Cell Differentiation During Visceral Leishmaniasis Infection

CD4^+^ T cell differentiation follows different pathways in human VL. When *L. donovani* parasites enters in human peripheral blood, CD4^+^ T cells (Th_0_) transformation started in response to pro inflammatory cytokines. Pro inflammatory cytokines released from antigen presenting cells play an important role in the conversion of Th_0_ cell into different lineages such as Th1, Th2, Th17 and Treg etc. Induction of IL-4/IL-13 cytokine signaling from antigen presenting cells induces the expression of GATA3 transcription factor which leads to conversion of Th_0_ cells into Th2 cells. Using potentially used webservers TargetScan, miRPath and miRDB web servers, we have identified putative miRNAs which subsequently down regulate the expression of transcription factor to control Th_0_ cell differentiation. In these web servers, we submitted the NCBI gene ID of key transcription factors and important genes to get the potential miRNAs.

## Results

### MiRNAs Regulating Th1/Th2 Differentiation and Plasticity

MiRNAs regulating Th1/Th2 signaling were predicted using three foremost web servers viz., TargetScan, (Agarwal et al., 2015) miRPath (Paraskevopoulou et al., 2013) and miRDB (Wong and Wang, 2015). TBX21 protein is a Th1 immune cell-specific transcription factor regulates Th1 immune signaling specific cytokines. By using TargetScan, we speculated that miR-29a, miR-29b, and miR-29c were found to be potential miRNA with the high probability of conserved targeting (P_CT_) value which can bind at position 245–251 of TBX21 3′-UTR. While, the miRNA obtained from miRPath and miRDB have shown that miR-548ah-5p, miR-526b-5p, and miR-4726-5p have the same target binding site at 3′-UTR of TBX21 gene. These miRNAs have the potential to down regulate the expression of TBX21 gene and subsequently inhibit the conversion of Th_0_ cells into Th1 cells. STAT1 and STAT4 transcription factors were also submitted for miRNA prediction since they play an important role in the development of Th1 cell and IFN-γ production. By using all three web servers, miR-1252, miR-4697, miR-4724, and miR-495 were found to be potential miRNA against STAT1 gene. Similarly, TargetScan and miRDB prediction have shown that miR-200a have the putative binding sites at position 237–244 of STAT4 gene with high P_CT_ values (**Table 3**). Entry of *L. donovani* parasites inside human peripheral blood induces a number of signaling in the CD4^+^ T cells which develops an environment to induce above miRNAs to negatively regulate Th1 immune cell conversion.

**Table 3:** MiRNAs regulating Th1 cell differentiation and plasticity.

Serial No.	Gene	miRDB	miRPath	TargetScan
1	TBX21	miR-548ah-5p miR-526b-5p miR-4726-5p	miR-548ah-5p miR-526b-5p miR-4726-5p	miR-29a miR-29b miR-29c
2	STAT1	miR-1252 miR-4697 miR-4724 miR-495	miR-1252 miR-4697 miR-4724 miR-495	miR-1252 miR-4697 miR-4724 miR-495
3	STAT4	miR-200a	–	miR-200a
4	IFNG	miR-24 miR-29	miR-24 –	miR-24 –
5	IL12	miR-21 miR-590-5p	miR-21 –	miR-21 miR-590-5p
6	RunX3	miR-130b miR-106b miR-130a	miR-130b miR-106b miR-130a	– – –
7	EOMES	miR-182 miR-29a miR-29b	miR-182 miR-29a miR-29b	miR-182 – –
8	IRF1	– – miR-4708-3p – miR-4483 –	miR-635 miR-4747-5p – miR-4667 – miR-23	miR-635 miR-4747-5p miR-4708-3p miR-4667 miR-4483 –

**FIGURE 1:**
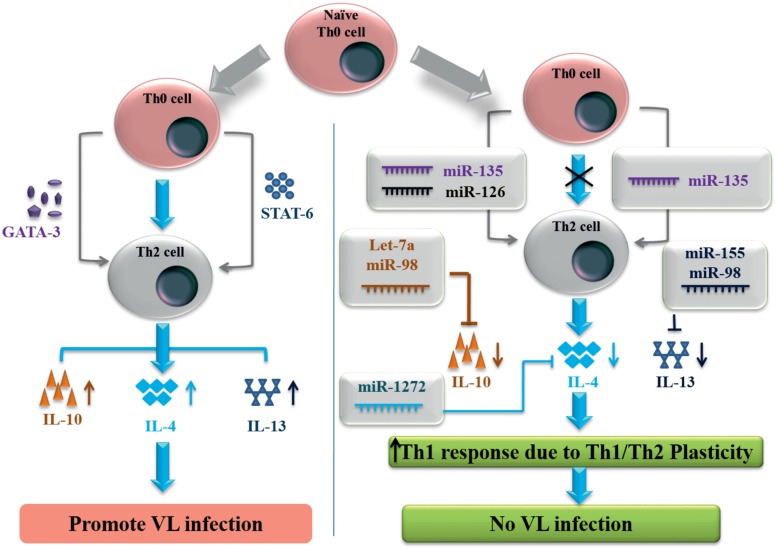
**MicroRNAs controlling Th2 signaling in human visceral leishmaniasis infection**.

Simultaneously, we have identified miRNAs against Th2 immune signaling molecules, which is responsible for *L. donovani* replication and VL disease development. GATA3 protein is T cell specific transcription factor which induces the conversion of Th_0_ into Th2 immune cells and IL-4/IL-13 signaling for developing a Th2 immune response during VL disease. GATA3 protein has multiple transcripts but for NM_001002295 transcript, by using all three web servers we found that miR-135 has the binding site at the 3′-UTR of GATA3 gene (position 207–213). MiR-135 has shown high P_CT_ value against GATA3 gene and has the potential to inhibit the expression of this gene and Th2 immune cell conversion. While miR-126 was predicted by miRPath and miRDB as a negative regulator of GATA3. STAT6 transcription factor induced by IL-4 has shown multiple transcripts with different 3′-UTR length. STAT6 gene with 3′-UTR length of 1168 nucleotides were found to show miR-135 binding at position 1100–1107. To control the VL infection, IL-4 and IL-13 pro-inflammatory cytokines released from antigen presenting cells can be inhibited by miR-1272 and miR-155 (**Figure 1**). Signature cytokines secreted by Th1/Th2 cell were also submitted for putative miRNA prediction, since they have important role in the maintenance of Th1/Th2 plasticity. MiRNAs against key transcription factor and important cytokines controlling Th1 and Th2 specific immune signaling events are listed in the **Tables 3** and **4** respectively.

**Table 4:** MiRNAs regulating Th2 cell differentiation and plasticity.

S. No.	Gene	miRDB	miRPath	TargetScan
1	GATA3	miR-135 miR-126	miR-135 miR-126	miR-135 -
2	IL4	miR-1272	miR-1272	miR-1272
3	STAT6	miR-135	-	miR-135
4	IL13	- - - - - -	let-7d let-7e let-7f let-7g let-7i miR-98	miR-155 - - - - -
5	IL10	- - - - - - -	miR-98 miR 4500 miR-4458 let-7a let-7b let-7c let-7d	miR-98 miR 4500 miR-4458 let-7a let-7b let-7c let-7d
6	IL2	miR-181a miR-181b miR-181c miR-181d	- - - -	miR-181a-5p miR-181b-5p miR-181c-5p miR-181d-5p
7	STAT5	mir-4663 - - - - - -	- miR-548k miR-3941 miR-4672 miR-4719 miR-524-5p miR-520d-5p	mir-4663 miR-548k miR-3941 miR-4672 miR-4719 miR-524-5p miR-520d-5p
8	IRF4	miR-128 miR-125b	miR-128 miR-125b	miR-128 -
9	GFI1	miR-142-3p	-	miR-142-3p
10	CMAF	- - -	miR-182 miR-143 miR-301a	miR-182 - -
11	JunB	miR-199a-5p miR-199b-5p	- -	miR-199a-5p miR-199b-5p

### MiRNAs Regulating Th17/Treg Differentiation and Plasticity

Subpopulations of CD4^+^ T cells producing IL-17 cytokines were capable to inducing IFN-γ in STAT4 dependent manner. During VL infection, it has been shown that IL-17 and IL-22 cytokine level increases in PBMC culture. Using TargetScan, miRPath and miRDB web servers, we have collected the series of miRNAs activated in response to *L. donovani* infection and controlling Th17/Treg differentiation. TargetScan and miRPath analysis have shown that miR-4500, let-7a, let-7b, and let-7c have the putative binding site at position 407-413 of 3′-UTR for RORC gene which can subsequently negatively regulate the expression of this gene for Th17 differentiation. RORC gene encodes for RORγt transcription factors. MiR-106a and miR-106b have shown putative binding site at the position 252–258 of 3′-UTR for STAT3 gene, when analyzed by all three web servers, which have the potential to down regulate the expression of this gene. MiR-124 was also found to be a negative regulator of STAT3 gene, by miRDB and miRPath and literature (Lu et al., 2013).

**FIGURE 2:**
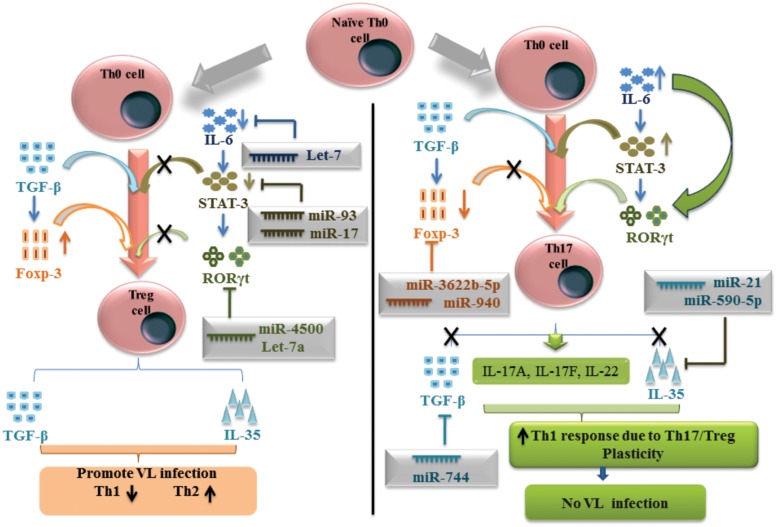
**MicroRNAs mediated plasticity between Thl7 and Treg cells in human visceral leishmaniasis**.

A small population of CD4^+^ T cells showing CD4^+^ CD25^+^ Foxp3^+^ characteristic termed as Treg cell and this subpopulation help in the *Leishmania* parasitic growth and development. TargetScan and miRDB prediction gave that miR-3622b-5p has shown putative binding site at position 796–802 of 3′-UTR for Foxp3 gene. MiR-940 and miR-1827 were predicted by TargetScan and miRPath, have shown binding site at position 413–419 and 305–315 respectively of 3′-UTR for the same gene, to down regulate the Treg-specific differentiation (**Figure 2**). Interestingly, all the predicted miRNAs for Foxp3 gene have shown different binding at 3′-UTR of Foxp3 gene. Th17/Treg plasticity is equally important for the *L. donovani* parasite to raise VL infection. In presence of Treg-specific miRNA environment, Th17 cell differentiation followed by IL-17 and IL-22 cytokine production, may further inhibit the growth of *L. donovani* parasites. Predicted miRNAs for the key transcription factors and important cytokines related to Th17 and Treg cells have been explained in the **Table 5**.

**Table 5:** MiRNAs regulating Th17 and Treg cell differentiation and plasticity.

Serial No.	Gene	miRDB	miRPath	TargetScan
1	RORC	- - - -	let-7a-5p let-7b-5p let-7c miR-4500	let-7a-5p let-7b-5p let-7c miR-4500
2	STAT3	miR-93 miR-519d miR-17 miR-106b miR-106a miR-124	miR-93 miR-519d miR-17 miR-106b miR-106a miR-124	miR-93 miR-519d miR-17 miR-106b miR-106a -
3	TGFβ	-	-	miR-744
4	IL6	-	-	let-7
5	Foxp3	miR-3622b-5p - -	- miR-940 miR-1827	miR-3622b-5p miR-940 miR-1827
6	IL35	- -	- -	miR-21 miR-590-5p

## Discussion

Human VL is associated with increased Th2 and Treg immune responses which induce the elevation of disease signature cytokines such as IL-6, IL-8, IL-10 and TGF-β. While a protective immune response against *L. donovani* is associated with elevated IFN-γ and IL-12 level, which leads to Th1 immune response. T cells developed from lymphoid progenitor stem cell in bone marrow and their maturation takes place in the thymus. MiRNAs have shown significant role in CD4^+^ T cell differentiation, maturation and to develop plasticity during cancer and infectious diseases (Liu et al., 2013). An immunological study indicates that Th1/Th2 paradigm is not solely responsible to controlling or favoring VL disease in the human model. Suppression of signature cytokines associated with Th1 immune response and elevation of Th2 type immune response cytokines have opened the window for development of immunotherapy against VL disease. Here, in this study we have predicted miRNAs as the regulatory molecule to control the CD4^+^ T cell differentiation to protect or favor VL disease. MiRNAs are an important player at the molecular level in cells which controls immune response in the human by controlling immune signaling at the post-transcriptional level. MiRNAs are emerging as a key controller for CD4^+^ T cell differentiation and for maintaining plasticity during VL infection. We targeted CD4^+^ T cell differentiation controlling transcription factor molecule for miRNA prediction which can regulate T cell differentiation phenomenon.

In our computational study, we observed that miR-548ah, miR-526b, miR-4726-5p, miR-29C, miR-29b, and miR-29a putatively control the expression of TBX21 protein expression in CD4^+^ T cell. During VL infection in Th2 type immune environment, negative regulation of TBX21 transcription factor by aforementioned miRNAs leads to raise the VL infection. Our computational study states that the elevated level of miR-1252, miR-4697, miR-4724, and miR-495, negatively regulates the STAT1 signaling. It was also noted that miR-200a expression in CD4^+^ T cells has the binding sites at the position 237-244 of 3′-UTR for STAT4 gene which can subsequently negatively regulate the Th1 type of immune response during VL infection (Huang et al., 2011).

In human VL infection, Th2 type of immune response activates to favor the replication of *L. donovani* in macrophages cells. Using TargetScan, miRPath and miRDB web servers, we identified miRNAs against the key transcription factor which has shown the potential to down regulate Th2 immune response. MiR-135 has a putative binding site to regulate GATA3 transcription factor which control the Th2 immune cell differentiation from CD4^+^ T cells. We also identified that miR-135 have the great potential to down regulate the expression of the STAT6 transcription factor at the post-transcriptional level in CD4^+^ T cells. Our finding indicates that let-7e, let-7c and miR-98 have the putative binding site at the 3′-UTR of IL-10 gene and negatively regulates the functional expression of IL-10. Our study states that miR-1272 and miR-155 have the potential to down regulate the IL-4/IL-13 signaling to check the Th2 response during VL infection. In the Th1 type of immune response micro-environment miR-1272 and miR-155 activated which subsequently inhibit the IL-4/IL-13 signaling pathway to suppress the Th2 type of immune response in human. Immune signaling polarization toward Th2 immune response is one of the main streams for the development of VL disease. In the presence of miRNAs investigated against GATA3, STAT6 transcription factors and IL-4, IL-13, IL-10 cytokines have the great potential to down regulate the Th2 immune response which subsequently induces the production of IFN-γ by Th1/Th2 plasticity phenomenon in human during VL infection. Furthermore, in the presence of elevated IFN-γ response, reactive oxygen species and nitric oxide signaling induces the parasitic clearance and protection from *L. donovani* parasites.

Mature T cells in peripheral blood of human consist of Th17 immune cells characterized by the production of IL-17, IL-21 and IL-22 cytokines. Protective role of IL-17 and IL-22 cytokines have been associated with the protection of human in response to *L. donovani* infection to suppress VL infection. Th17 immune cell differentiation from CD4^+^ T cell takes place by the expression of ROR-γt transcription factor. MiR-4500 and let-7c were found to have a putative binding site at the 3′-UTR of RORC gene, which negatively regulates the maturation and differentiation of Th17 cells during VL infection. In the condition of elevated expression of miR-93 and miR-124, miR-106a, and miR-106b, Th17 immune diminished and *L. donovani* parasite finds the favorable condition to replicate in the macrophages of human. Regulatory T cells are the subpopulation of CD4^+^ T cells and characterized by the expression of CD4^+^ CD25^+^ Foxp3^+^ in human VL and associated with the production of TGF-β cytokines to favor the parasite replication in VL disease. Our study indicates that miR-744 suppresses the expression of TGF-β cytokine which subsequently have the potential to inhibit the Treg cell differentiation and maturation in VL disease (Butz et al., 2012).

Our study highlights the importance of miRNAs in the CD4^+^ T cell differentiation, maturation and their functional aspects toward the development of protection from *L. donovani* parasites. This computational study generates the signaling mechanism controlled and regulated by miRNAs in human VL infection. MiRNA developed against Th2 and Treg immune cells are important and their over expression can be used for developing Th1 and Th17 type of protection specific immune response during VL infection. In cancer disease, many miRNA based therapy is under clinical trial and have shown excited efficacious response by developing a protective immune response. By this study, we have postulated that miRNAs against key transcription factors and important cytokines can be used to design miRNA based therapy to develop Th1 and Th17 type of protective immune response to generate IFN-γ and nitric oxide signaling to kill *L. donovani* parasites. By understanding miRNA regulatory network to control CD4^+^ T cell differentiation, we can develop less toxic accurate and targeted therapy against VL disease. By this fascinating bioinformatics based study one can provide the intricate mechanism to control CD4^+^ T cell differentiation and to develop gene regulation mechanism to inhibit *L. donovani* growth and replication. This study further develops the deep concern for the development of miRNA based therapy in VL disease.

## Author Contributions

Conceived and designed the experiments: RKP, SS, and VKP. Performed the experiments: RKP and VKP. Analyzed the data: RKP and VKP. Contributed reagents/materials/analysis tools: RKP and VKP. Wrote the paper: RKP, SS, and VKP.

## Conflict of Interest Statement

The authors declare that the research was conducted in the absence of any commercial or financial relationships that could be construed as a potential conflict of interest.
